# HARMONY Study: Pimavanserin Significantly Prolongs Time to Relapse of Dementia-Related Psychosis

**DOI:** 10.1093/geroni/igaa057.530

**Published:** 2020-12-16

**Authors:** Pierre Tariot, Erin P Foff, Jeffrey L Cummings, Maria-Eugenia Soto-Martin, Bradley McEvoy, Srdjan Stankovic, Amy Howard

**Affiliations:** 1 Banner Alzheimer’s Institute, Pheonix, Arizona, United States; 2 ACADIA Pharmaceuticals Inc., Princeton, New Jersey, United States; 3 Cleveland Clinic Lou Ruvo Center for Brain Health, Las Vegas, Nevada, United States; 4 Gerontopole Alzheimer Clinical Research Center/University Hospital of Toulouse, Toulouse, France; 5 ACADIA Pharmaceuticals Inc., San Diego, California, United States

## Abstract

Dementia-related psychosis (DRP) is common among patients with Alzheimer’s disease (AD), Parkinson’s disease (PD), dementia with Lewy bodies (DLB), frontotemporal dementia (FTD), and vascular dementia (VaD) and is associated with poor outcomes. HARMONY (NCT03325556) was a Phase 3, placebo-controlled, randomized, relapse-prevention study evaluating the efficacy and safety of pimavanserin for treating hallucinations and delusions associated with DRP. Patients with dementia and moderate-severe psychosis received open-label (OL) pimavanserin for 12 weeks. Patients with sustained response (≥30% reduction in Scale for the Assessment of Positive Symptoms hallucinations+delusions Total Score AND Clinical Global Impression-Improvement score of much/very much improved) at Weeks 8 and 12 were randomized 1:1 to continue pimavanserin or receive placebo for up to 26 weeks in the double-blind (DB) period. The primary endpoint was time from randomization to relapse of DRP. 392 patients enrolled. 217 (61.8%) eligible patients experienced sustained response and were randomized. OL response was similar regardless of dementia subtype (randomization rates: 59.8% AD, 71.2% PDD, 71.4% VaD, 45.5% DLB, 50.0% FTD), baseline disease characteristics, age, dementia severity, or previous drug therapy. The study stopped early for superior efficacy when a prespecified interim analysis revealed >2.8-fold reduction in risk of relapse with pimavanserin (hazard ratio: 0.353; 95% CI: 0.172, 0.727; 1-sided p=0.0023). Adverse event rates were low and balanced (OL: 36.2%; DB: 41.0% pimavanserin, 36.6% placebo). No negative impact on cognition or motor function was observed. The Harmony study demonstrated a robust decrease in hallucinations and delusions and significant maintenance of efficacy with pimavanserin treatment in DRP.

