# Erratum to: HARMONY Study: Pimavanserin Significantly Prolongs Time to Relapse of Dementia-Related Psychosis

**DOI:** 10.1093/geroni/igab011

**Published:** 2021-05-04

**Authors:** Pierre Tariot, Erin P Foff, Jeffrey L Cummings, Maria-Eugenia Soto-Martin, Bradley McEvoy, Srdjan Stankovic

**Affiliations:** 1 Banner Alzheimer’s Institute, Pheonix, Arizona, United States; 2 ACADIA Pharmaceuticals Inc., Princeton, New Jersey, United States; 3 Cleveland Clinic Lou Ruvo Center for Brain Health, Las Vegas, Nevada, United States; 4 Gerontopole Alzheimer Clinical Research Center/University Hospital of Toulouse, Toulouse, France; 5 ACADIA Pharmaceuticals Inc., San Diego, California, United States

In the published abstract, “HARMONY STUDY: PIMAVANSERIN SIGNIFICANTLY PROLONGS TIME TO RELAPSE OF DEMENTIA-RELATED PSYCHOSIS,” doi: 10.1093/geroni/igaa057.530” Amy Howard was erroneously listed as a co-author. The author list has since been corrected to: Pierre Tariot, Erin P. Foff, Jeffrey L. Cummings, Maria-Eugenia Soto-Martin, Bradley McEvoy, and Srdjan Stankovic.

